# Comparison of different imaging devices and navigation systems for cervical pedicle screw placement: an experimental study on screw accuracy, screw placement time and radiation dose

**DOI:** 10.1038/s41598-024-77191-2

**Published:** 2024-11-12

**Authors:** Eric Mandelka, Justine Wolf, Antonia Medrow, Paul A. Gruetzner, Sven Y. Vetter, Jula Gierse

**Affiliations:** 1 Research group Medical Imaging and Navigation in Trauma and Orthopedic Surgery (MINTOS), Department of Orthopedics and Trauma Surgery, BG Klinik Ludwigshafen, Ludwig- Guttmann-Str. 13, 67071 Ludwigshafen, Germany; 2 Heidelberg University, Grabengasse 1, 69117 Heidelberg, Germany

**Keywords:** Cervical spine, Cervical pedicle screws, Posterior screw fixation, Spinal navigation, Intraoperative 3D imaging, Radiation dose, Bone, Trauma, Fracture repair, Bone imaging, Three-dimensional imaging, Experimental models of disease

## Abstract

Cervical pedicle screws (CPS) provide biomechanically superior fixation compared to other techniques but are technically more demanding. Navigated CPS placement has been increasingly reported as a safe and accurate technique, yet there are few studies comparing different combinations of imaging and navigation systems under comparable conditions. With this study, we aimed to compare different imaging and navigation systems for CPS placement in terms of accuracy, screw placement time and applied radiation dose. For this experimental study, navigated CPS placement was performed at levels C2 to C7 in 24 identical radiopaque artificial spine models by two surgeons with different levels of experience using three different combinations of intraoperative 3D imaging devices and navigation systems. Accuracy, time and radiation dose were compared between the groups. In total, 288 screws were placed. Accuracy was > 98% in all groups with no significant differences between groups or between surgeons (*P* = 0.30 and *P* = 0.31, respectively), but the inexperienced surgeon required significantly more time (*P* < 0.001). Radiation dose was significantly higher with iCT compared to CBCT (*P* < 0.0001). Under experimental conditions, accuracy rates of > 98% were achieved for navigated CPS placement regardless of the imaging modality or navigation system used. Radiation doses were significantly lower for CBCT compared to iCT guidance.

## Introduction

Cervical pedicle screw (CPS) placement was first described for traumatic injuries by Abumi et al. in 1994^1^. Meanwhile, numerous studies have demonstrated clinical feasibility of CPS placement as well as its biomechanical superiority over alternative cervical fixation methods for numerous indications^2–6^. Nevertheless, compared to pedicle screw placement in the thoracic and lumbar spine, the number of studies on cervical pedicle screws is limited. The clinical application of CPS is rather rare compared to lateral mass screws (LMS) since CPS placement is more challenging due to close proximity of the pedicle to neurovascular structures^3,7–11^. In addition to the variable anatomy of cervical pedicles, the high mobility of the cervical spine also entails challenging surgical conditions^7,12–15^. The pedicle as the screw corridor is only a few millimeters wide, leaving limited room for error^1,16^. Although not all pedicle wall perforations cause complications and a margin of acceptable error is considered in different classification systems rating CPS accuracy, surgeons should always aim to avoid perforations of the pedicle when placing screws^17–19^. Yet, perforation rates of 3.8–27% have been reported for freehand and fluoroscopy-guided screw placement^20–22^. Since the introduction of intraoperative navigation based on intraoperative computed tomography (iCT) or cone beam computed tomography (CBCT), various studies have compared fluoroscopy-guided and navigation-based techniques and have shown significantly greater accuracy rates and less postoperative complications for navigated screw placement^17,23–25^. In addition, this technique can reduce the time needed for surgery and radiation exposure to staff^26,27^. However, the number of studies investigating different combinations of 3D imaging devices and navigation systems for navigated CPS placement is scarce. Therefore, the aim of this study was to compare three different combinations of imaging devices and navigation systems regarding accuracy of cervical pedicle screw placement, radiation dose as well as the time needed for screw placement.

## Methods

**Fig. 1:**
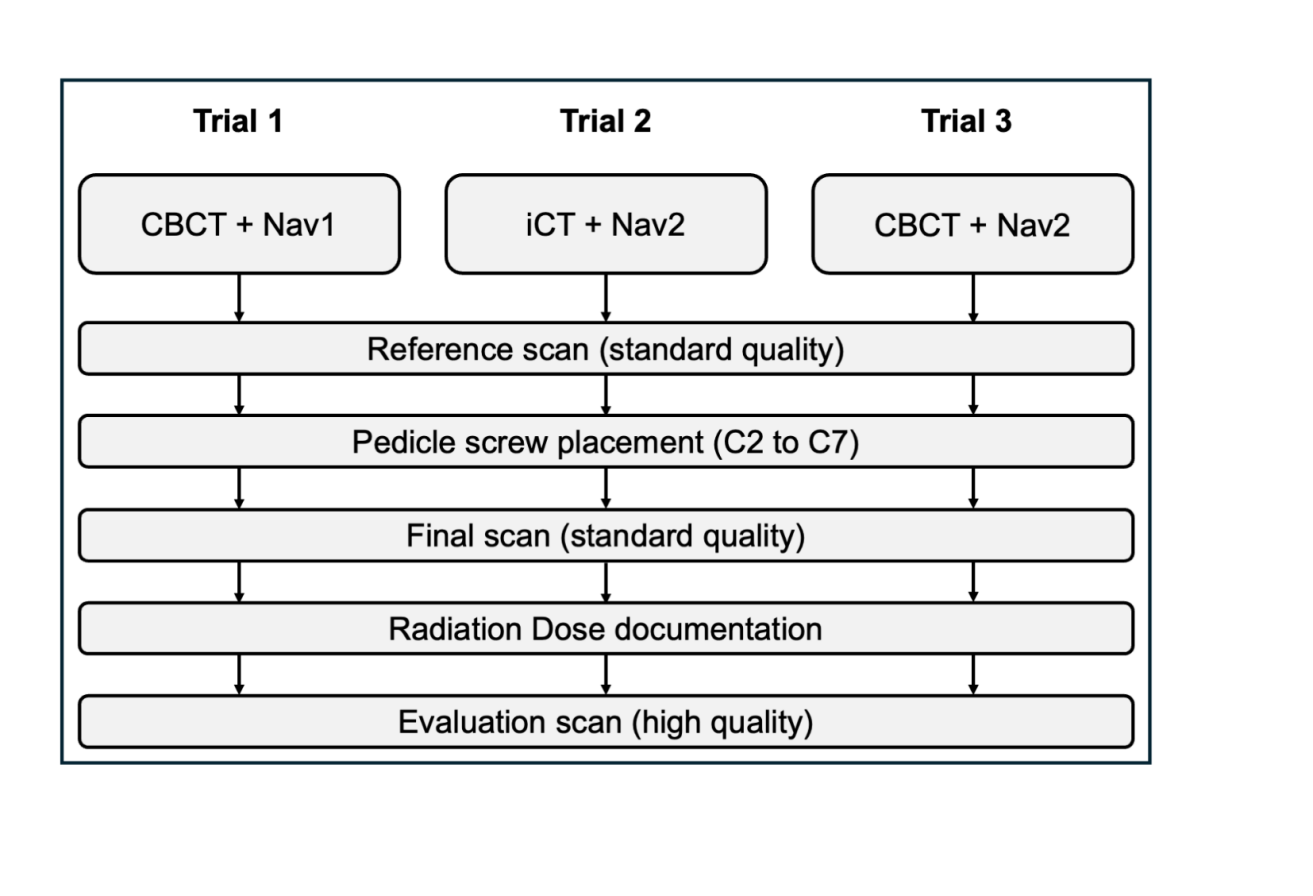
Study design.

In this experimental study, CPS placement was performed on 24 identical radiopaque artificial cervical spine models (Synbone, Zizers, Switzerland). Three different combinations of intraoperative imaging devices and navigation systems were used. Investigated were a 3D C-arm CBCT (cCBCT; Cios, Siemens Healthineers, Erlangen, Germany) in combination with Nav1 (Pulse, NuVasive, San Diego, California, USA) navigation software (Trial 1). The second combination investigated was a mobile iCT (Airo, Brainlab, Munich, Germany) and Nav2 (Curve Image Guided Surgery, Brainlab, Munich, Germany) (Trial 2). In the third trial, the combination cCBCT and Nav2 was investigated (Trial 3; Fig. 1).

In each of the 24 models, 12 screws were inserted bilaterally in levels C2 to C7. Depending on the navigation system, the following two respective screw systems, were used: the VuePoint system (NuVasive, San Diego, California, USA) in Trial 1 and the Symphony system (DePuy Synthes, Raynham, Massachusetts, USA) in Trial 2 and 3. All screws placed were 3.5 mm in diameter. Screw length was determined depending on vertebral size of the respective cervical spine level. All screw sizes used were identical for both screw systems, as all models were identical. The screws were placed by two different surgeons, an experienced spine surgeon (ES) prone to navigated procedures and a third-year resident surgeon (RS) with no experience in navigated screw placement or spine surgery in general. In every trial, each surgeon placed screws unilaterally in an open approach. The surgeons aimed for most accurate screw placement possible.

To perform CPS placement, the models were placed in prone position in a spine bed (Synbone, Zizers, Switzerland). The spine bed was then placed on a radiolucent carbon table and fixed to the table with tension belts to avoid movement of the model. The dynamic reference base (DRB) of the navigation system was mounted on the spinous process of C5. A scan of the model was obtained using the respective imaging device and transferred to the navigation system. Using the cCBCT (Trial 1 and 3), the reference scan was acquired in standard quality (200 images, 30 s). In Trial 2 the reference was performed using the standard settings for imaging of the cervical spine (adult cervical spine, 120.0 kV; 95.0 mA). Navigation instruments were then calibrated, and the accuracy of image registration was verified at different anatomical landmarks using the navigated pointer. Subsequently, screw placement was initiated: The navigated drill guide was used to locate the entry point and trajectory of the screw, which was then saved to the navigation system. Trajectory was continuously controlled while electric drilling was performed with a preset depth of 20 mm. Finally, immediately after drilling the screw hole, the screw was placed according to the planned trajectory using the navigated screwdriver. In all models, screw placement was performed from C2 to C7 unilaterally before the contralateral side was instrumented. After every single screw placed the navigation accuracy was checked using the navigated instruments to prevent inaccuracies caused by the torque of previous screw placement.

Screw placement time was defined as the time required to place a screw from the first contact of the drill guide with the pedicle of the spine model until the screw was placed to the surgeon’s satisfaction. Screw placement time did not include the time needed for image acquisition and calibration of the navigated instruments. After all screws were placed, a second standard quality scan (200 images, 30 s) was performed, according to the clinical procedure. This scan was considered in the radiation measurement. Subsequently, for the purpose of this study and to allow optimal assessment of screw position, another scan was performed in high quality. Assessment of cervical pedicle screw position was performed by a blinded observer according to the Neo classification and repeated after six weeks^28^. As suggested by Neo et al., screws with no breach (Grade 0) or a breach of < 2 mm (Grade 1) were deemed acceptable while breaches of ≥ 2 mm (Grade 2–3) were considered inacceptable.

All screws were assessed twice by the observer. If a screw was rated differently in the two evaluations, the worse rating was used.

To compare the radiation dose applied during navigated screw placement between the groups, the dose protocols were retrieved of the two imaging systems used. To allow comparability between the dose area product (DAP; [mGycm^2^) as reported by the 3D C-arm CBCT and the dose length product (DLP; [mGycm]) as reported by the iCT, the data was converted into effective dose ([mSv]) using conversion factors. For DAP, a conversion factor of 0.27 mSv/mGycm^2^ was used as suggested by Hwang et al.^29^. For DLP, a conversion factor of 0.0059 mSv/mGycm was used as suggested by the American Association of Physicists in Medicine for the anatomical region of the neck.

Kolmogorov-Smirnov test was used to check for normal distribution of data. Descriptive statistics for continuous variables are shown as means ± standard deviation (SD) with Gauss distribution, and median ± 95% confidence interval (95% CI) for non-normally distributed data.

The statistical tests were chosen according to the distribution of the data and the assumptions of the tests: Screw accuracy according to the Neo classification was compared between the trials using Kruskal-Wallis test with Dunn’s correction for multiple comparisons. Two-tailed Fisher’s exact test was used to compare screw accuracy rates between surgeons. Screw placement, reported in minutes (min), between the trials was compared using Kruskal-Wallis test with Dunn’s correction for multiple comparisons. Two-tailed Mann-Whitney test was chosen for comparison of time demand between the surgeons. The radiation dose measured was compared between trials using ordinary one-way ANOVA with Tukey’s correction for multiple comparisons.

To calculate intrarater reliability, kappa (κ) was used with its interpretation according to Landis and Koch. To account for close matches of the two assessments, Kappa was weighted (κ_w_).

The screw-to-pedicle diameter ratio was calculated for all levels. The pedicle diameters were measured in an iCT scan performed prior to screw placement. After multiple measurements at each level, the smallest diameter perpendicular to the optimal screw axis was considered the pedicle diameter.

The significance level was set at *P* < 0.05. Prism 10 (Version 10.1.1, Graphpad Software, San Diego, California, USA) was used for statistical analysis and figure creation.

## Results

**Fig. 2:**
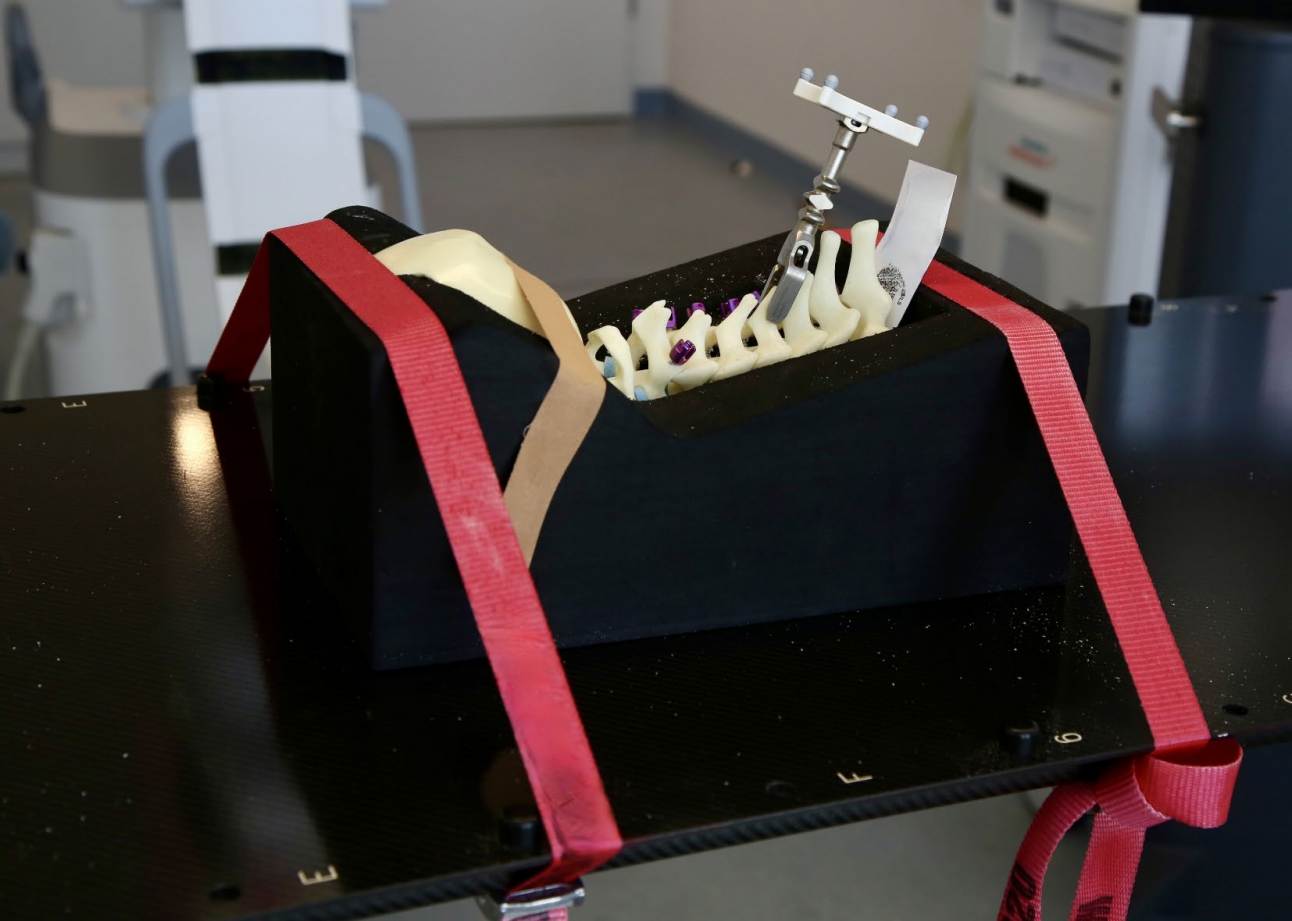
Oblique view on the cervical spine model with a lateral Grade 1 perforation of C5.

**Fig. 3:**
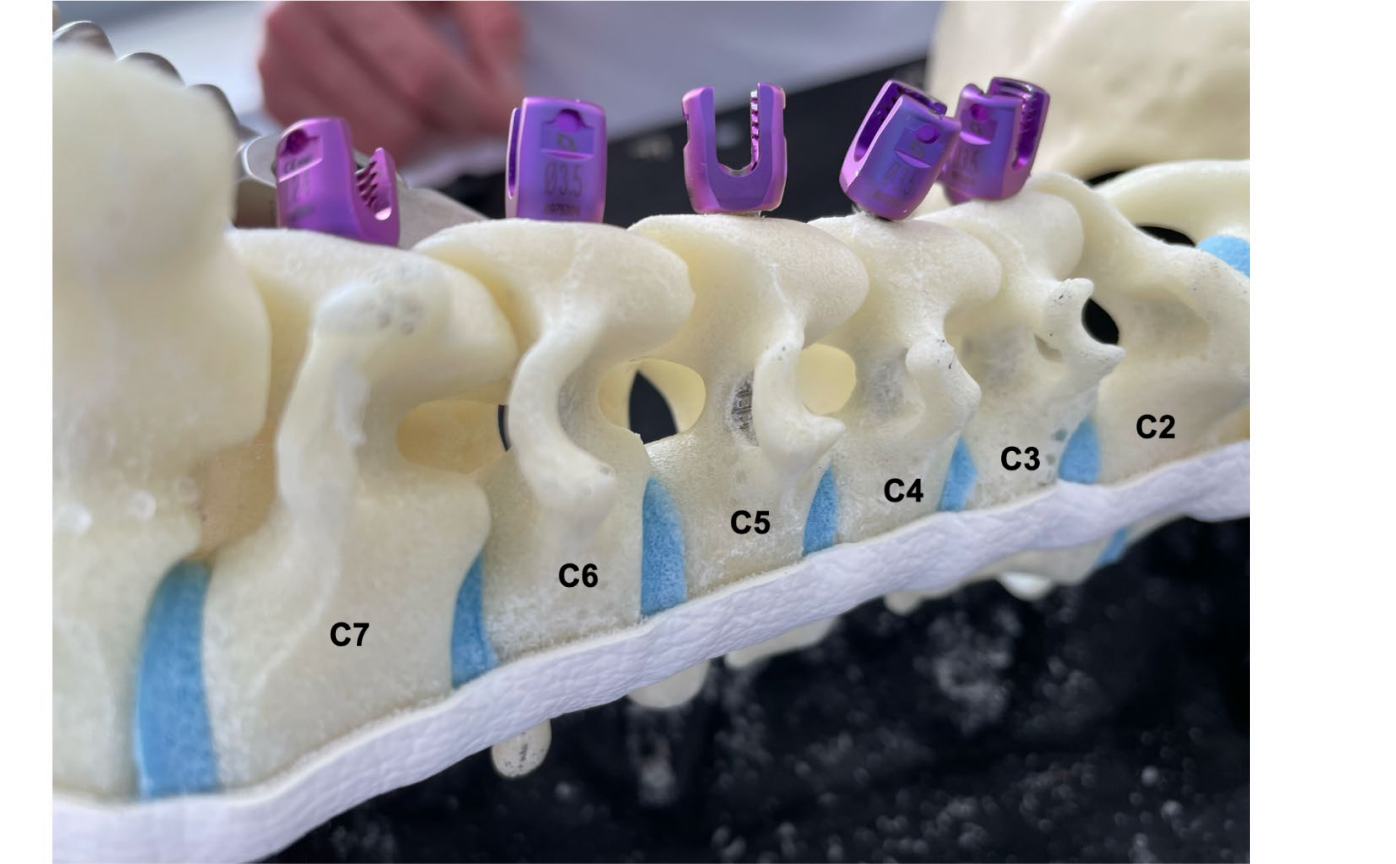
Accuracy of CPS placement according to Neo classification for Trials 1, 2 and 3 (*n* = 96 each).

80 subaxial pedicle screws and 16 C2 pedicle screws were placed in each trial, resulting in a total of 288 screws placed (Fig. 2). The overall screw placement accuracy was 99.1% for Trial 1, 99.1% for Trial 2 and 98.2% for Trial 3 showing no statistically significant differences (*P* = 0.30; Fig. 3). The comparison of accurately placed screws depending on the surgeon also did not show a significant difference with 97.9% and 99.3% accuracy for the RS and the ES, respectively (*P* = 0.31). One critical perforation each was found for C3, C4, C6 as well as C7 (Table 1). Except for C7, all critical perforations occurred on the lateral pedicle wall. The screw-to-pedicle diameter ratio for the levels C2 to C7 is shown in Table 2.

**Table 1:** Neo classification Grade considering trial, surgeon and vertebral level.

Level	Grade	Trial 1				Trial 2				Trial 3			
		ES		RS		ES		RS		ES		RS	
		*n*	(%)	*n*	(%)	*n*	(%)	*n*	(%)	*n*	(%)	*n*	(%)
C2	0	3	(37.5)	1	(12.5)	4	(50.0)	3	(37.5)	4	(50.0)	0	(0.0)
	1	5	(62.5)	7	(87.5)	4	(50.0)	5	(62.5)	4	(50.0)	8	(100.0)
	2	0	(0.0)	0	(0.0)	0	(0.0)	0	(0.0)	0	(0.0)	0	(0.0)
	3	0	(0.0)	0	(0.0)	0	(0.0)	0	(0.0)	0	(0.0)	0	(0.0)
Total Accuracy		**8**	**(100.0)**	**8**	**(100.0)**	**8**	**(100.0)**	**8**	**(100.0)**	**8**	**(100.0)**	**8**	**(100.0)**
C3	0	0	(0.0)	0	(0.0)	0	(0.0)	0	(0.0)	0	(0.0)	0	(0.0)
	1	8	(100.0)	8	(100.0)	8	(100.0)	8	(100.0)	7	(87.5)	8	(100.0)
	2	0	(0.0)	0	(0.0)	0	(0.0)	0	(0.0)	1	(12.5)	0	(0.0)
	3	0	(0.0)	0	(0.0)	0	(0.0)	0	(0.0)	0	(0.0)	0	(0.0)
Total Accuracy		**8**	**(100.0)**	**8**	**(100.0)**	**8**	**(100.0)**	**8**	**(100.0)**	**7**	**(87.5)**	**8**	**(100.0)**
C4	0	0	(0.0)	0	(0.0)	0	(0.0)	0	(0.0)	0	(0.0)	0	(0.0)
	1	8	(100.0)	8	(100.0)	8	(100.0)	8	(100.0)	7	(87.5)	8	(100.0)
	2	0	(0.0)	0	(0.0)	0	(0.0)	0	(0.0)	1	(12.5)	0	(0.0)
	3	0	(0.0)	0	(0.0)	0	(0.0)	0	(0.0)	0	(0.0)	0	(0.0)
Total Accuracy		**8**	**(100.0)**	**8**	**(100.0)**	**8**	**(100.0)**	**8**	**(100.0)**	**7**	**(87.5)**	**8**	**(100.0)**
C5	0	1	(12.5)	2	(25.0)	0	(0.0)	0	(0.0)	1	(12.5)	2	(25.0)
	1	7	(87.5)	6	(75.0)	8	(100.0)	8	(100.0)	7	(87.5)	6	(75.0)
	2	0	(0.0)	0	(0.0)	0	(0.0)	0	(0.0)	0	(0.0)	0	(0.0)
	3	0	(0.0)	0	(0.0)	0	(0.0)	0	(0.0)	0	(0.0)	0	(0.0)
Total Accuracy		**8**	**(100.0)**	**8**	**(100.0)**	**8**	**(100.0)**	**8**	**(100.0)**	**8**	**(100.0)**	**8**	**(100.0)**
C6	0	0	(0.0)	0	(0.0)	1	(12.5)	0	(0.0)	2	(25.0)	1	(12.5)
	1	8	(100.0)	8	(100.0)	7	(87.5)	7	(87.5)	6	(75.0)	7	(87.5)
	2	0	(0.0)	0	(0.0)	0	(0.0)	1	(12.5)	0	(0.0)	0	(0.0)
	3	0	(0.0)	0	(0.0)	0	(0.0)	0	(0.0)	0	(0.0)	0	(0.0)
Total Accuracy		**8**	**(100.0)**	**8**	**(100.0)**	**8**	**(100.0)**	**7**	**(87.5)**	**8**	**(100.0)**	**8**	**(100.0)**
C7	0	0	(0.0)	2	(25.0)	3	(37.5)	4	(50.0)	1	(12.5)	0	(0.0)
	1	7	(87.5)	6	(75.0)	5	(62.5)	4	(50.0)	7	(87.5)	8	(100.0)
	2	1	(12.5)	0	(0.0)	0	(0.0)	0	(0.0)	0	(0.0)	0	(0.0)
	3	0	(0.0)	0	(0.0)	0	(0.0)	0	(0.0)	0	(0.0)	0	(0.0)
Total Accuracy		**7**	**(87.5)**	**8**	**(100.0)**	**8**	**(100.0)**	**8**	**(100.0)**	**8**	**(100.0)**	**8**	**(100.0)**

**Table 2:** Screw and pedicle diameter as well as the corresponding screw-to-pedicle diameter ratio (SPR) for all levels.

Level	Diameter [mm]		SPR
	Screw	Pedicle	
C2	3.5	4.3	0.81
C3	3.5	3.5	1.00
C4	3.5	3.2	1.10
C5	3.5	3.3	1.06
C6	3.5	4.3	0.81
C7	3.5	5.5	0.64

**Fig. 4:**
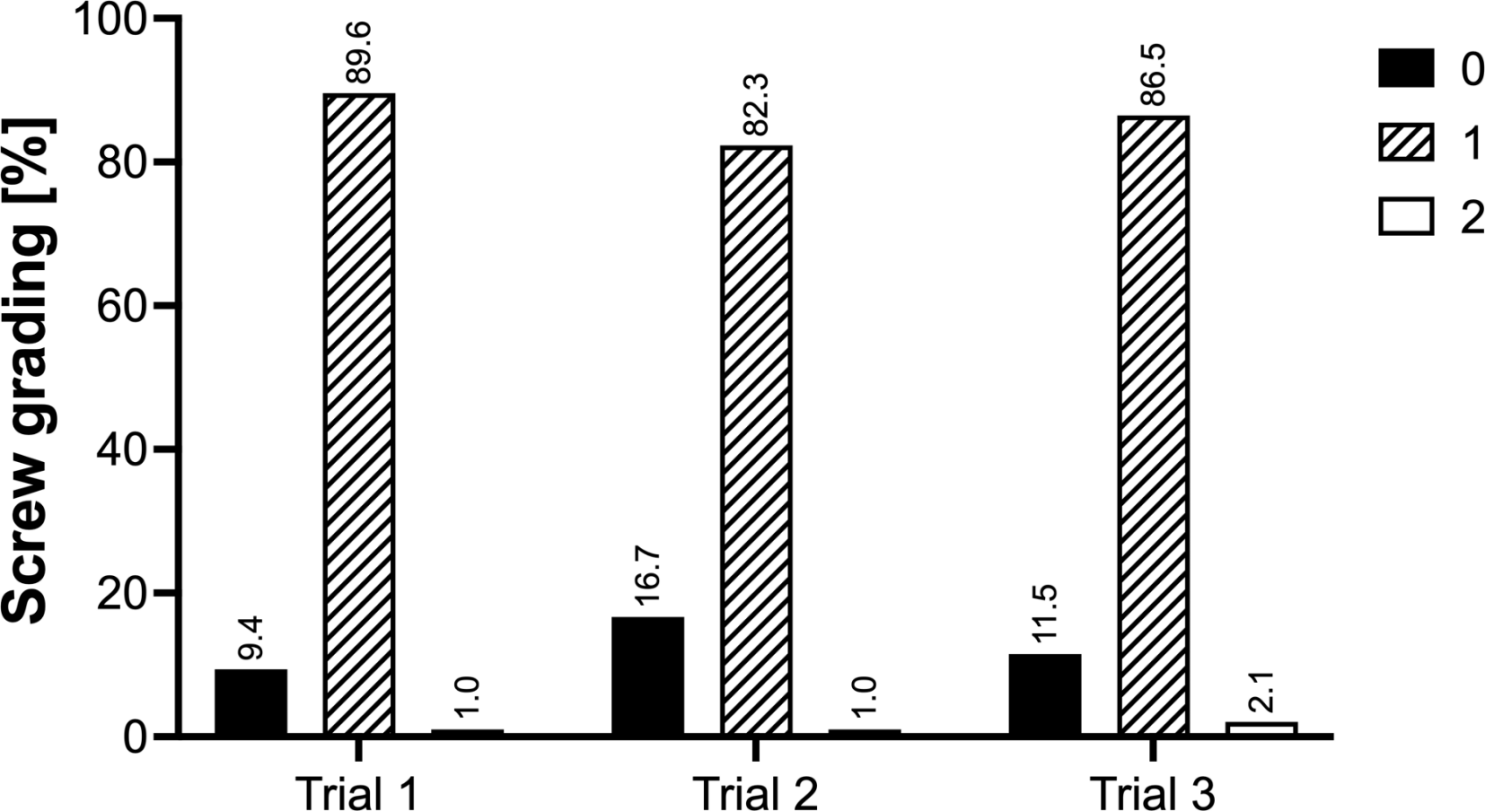
Weighted intra-rater reliability κ ± 95% confidence interval for the exact grading according to the Neo classification for the different imaging modalities.

Intra-rater agreement was fair for all imaging modalities with exact grading agreement in 70 (72.9%), 65 (65.7%) and 67 cases (69.8%), for Trial 1, Trial 2 and Trial 3, respectively (Fig. 4). The agreement on critical perforations was 95/96 (99.0%) for Trial 1 and Trial 3 and 96/96 (100.0%) for Trial 2.

Screw placement time per screw was significantly longer in Trial 1 (1.97 min; 95% CI [1.80, 2.30]) compared to Trial 2 (1.17 min; 95% CI [1.07, 1.33]; *P* < 0.0001) and Trial 3 (1.35 min; 95% CI [1.22, 1.48]; *P* < 0.0001) while the comparison of Trials 2 and 3 showed no significant difference (*P* = 0.77). Statistical analysis of the screw placement time showed a significant difference between the experienced spine surgeon (ES) and the resident surgeon (RS) with a median of 1.32 min (95% CI [1.37, 1.68]) for the ES compared to a median of 2.10 min (95% CI [1.78, 2.40]) for the RS (*P* < 0.001). The difference between the surgeons was significant for Trial 1 (*P* < 0.001) and Trial 3 (*P* = 0.04), while the difference for Trial 2 did not reach significance (*P* = 0.05). For the RS, a learning curve was observed during Trial 1 when comparing the time needed for placement of the first 24 screws compared to the following 24 screws with a median time of 3.1 min (95% CI [2.7, 5.5]) compared to 2.0 min (95% CI [1.8, 2.8]; *P* = 0.006).

**Fig. 5:**
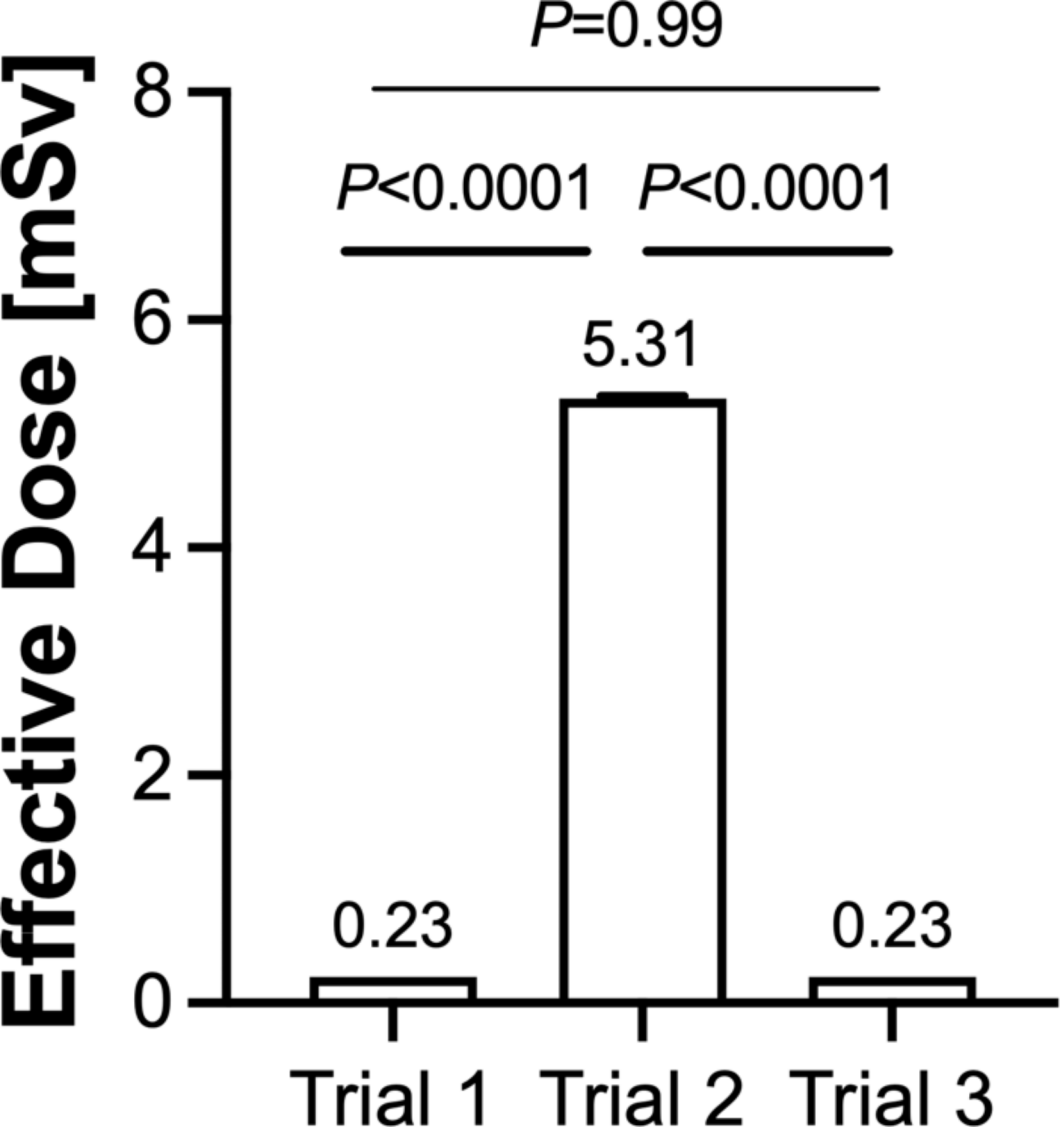
Mean effective dose per model in each group (*n* = 8 each).

The mean radiation dose measured in the different trials is shown in Fig. 5. Statistical analysis showed a significant difference between Trials 1 and 2 (*P* < 0.0001) as well as Trials 2 and 3 (*P* < 0.0001), while no significant difference between Trials 1 and 3 was found (*P* = 0.99).

## Discussion

The aim of this study was to compare three different combinations of intraoperative imaging devices and navigation systems for CPS placement under standardised experimental conditions. In addition to the accuracy of cervical pedicle screw placement, the screw placement time and the radiation dose applied were measured.

The results of this study show high accuracy rates of over > 98% for navigated cervical pedicle screw placement regardless of the imaging modality or navigation system used despite the high screw-to-pedicle diameter ratio – especially for the levels C3, C4 and C5.

These results add to previous reports on the accuracy of cervical pedicle screw placement using different techniques, such as fluoroscopic guidance and spinal navigation^23,24,30–33^. In their systematic review of 1,427 CPS placed under clinical conditions using navigation, Mahmoud et al. reported an average accuracy rate of 91.4%^23^. In another review, Tarawneh et al. found relevant pedicle perforations to be present in 12.5% of screws^24^. Using CBCT-based navigation (O-arm and StealthStation S7 navigation system, both Medtronic, Minneapolis, Minnesota, USA), Chachan et al. as well as Ishikawa et al. reported accuracy rates of approximately 95%^20,30^. Both studies used the Gertzbein-Robbins grading system to assess screw position, which is comparable to the Neo classification used in the present study^34^. However, none of the above studies compared cervical pedicle screw placement using different imaging and navigation systems under quasi-identical conditions.

The differences between the results presented and those reported by other authors may be due to the limited comparability of the experimental setup with clinical reality and the use of different imaging devices and navigation systems. In addition, the surgeons in this study had complete visual feedback due to the open spine model setup, which may have influenced the accuracy screw placement.

In contrast to our clinical procedure, in which the screws are placed only after all drill holes have been completed and the corresponding screw trajectories have been marked with K-wires, in this study, due to the lack of cannulated screw systems, the screw was placed immediately after drilling. In clinical reality, this technique should be avoided in order to avoid a potential compromise of navigation accuracy due to a change in anatomical configuration. In our experimental setup, the navigated instruments were used to check the navigational accuracy using anatomical landmarks after each screw.

Although several studies have shown advantages of navigated over other CPS placement techniques, freehand and fluoroscopically guided techniques are widely used. Jung et al. postulated that the use of a freehand technique without image-guidance may result in screw placement accuracy comparable to image-guided techniques when performed by a well-trained surgeon. The authors concluded that regardless of the technique used, surgeon experience is the determining factor in screw accuracy^21^. In contrast, for navigated screw placement, the results of the present study show no significant differences in screw placement accuracy between an experienced spine surgeon and an inexperienced resident. However, it should be considered that real-life intraoperative factors did not interfere with CPS placement in this study due to its experimental design and the use of artificial bone models. Nevertheless, the results presented suggest that navigation in general may be a helpful tool for the less experienced surgeon and could be used to improve the accuracy of screw placement. While there was no difference in accuracy, the inexperienced surgeon took significantly more time to place the screws than the experienced spine surgeon. However, this difference may not be clinically relevant as the average difference, although significant, was approximately 30 s per screw. In addition, the inexperienced surgeon showed a learning curve regarding the time required for CPS placement, as reported by Heo et al.^35^. This may also be the reason for the significantly longer screw placement time in Trial 1 compared to Trials 2 and 3.

However, experience in spine surgery, and in particular cervical spine surgery, and in the use of intraoperative navigation is crucial to minimise the challenges of navigated CPS placement. In particular, the cervical spine has a high degree of intersegmental mobility, which is a major concern when using navigation in this region, as it can lead to a mismatch between the actual and displayed anatomy. A further challenge is the selection of a sufficiently stable position for the Dynamic Reference Base (DRB). This is essential to avoid its interference with the surgical field, which could lead to navigation inaccuracies due to unintentional contact with the array, and also to minimise the relative movements of the instrumented vertebra in relation to the vertebra on which the array is mounted^36,37^. This risk was unequal between the navigation systems, as the DRB of Nav2 was evidently smaller than that of Nav1. However, considering the results presented, this inequality did not seem to effect accuracy rates in this study.

Although the absolute numbers of radiation doses measured cannot be compared to clinical practice, this experimental study showed significant differences between the imaging devices investigated. The effective doses applied in Trials 1 and 3 were comparable, which was to be expected as the same intraoperative imaging system was used in both trials. In Trial 2, in which the iCT was used, the effective dose was more than 23 times higher compared to Trial 1 and 3. When comparing different imaging systems regarding radiation dosage, previous studies have shown inconsistent results^38,39^. A recent study performed by Klingler et al. reported the radiation dose for intraoperative imaging of the cervical spine using iCT to be approximately 14 times higher when measured at the thyroid gland and 42 times higher when measured at the eye lens of the patient compared to 3D C-arm CBCT, which is in accordance with the results presented here^40^.

Tkatschenko et al. have stated that the potential benefits of 3D navigation may outweigh the potentially higher one-time radiation exposure of 3D imaging compared to fluoroscopy; however, minimising radiation exposure to the patient and staff should remain a priority, especially when comparable systems with much lower radiation doses are available^41^.

A comparison of radiation exposure for lumbar screw placement using the same imaging devices was performed on human specimens by Foster et al. Interestingly, the authors found no significant difference between the iCT and CBCT, which may be due to a different form of data collection and a different spinal region investigated^42^.

The main limitation of this study is the use of artificial spine models in an experimental setting. Due to the experimental setup and the lack of soft tissue in our setup, the absolute radiation doses measured cannot be compared to doses recorded in a clinical setting. However, creating quasi-identical conditions to allow direct comparability, as seen in this study, in a clinical setting is challenging, if not impossible. Other considerations when using spinal models include the lack of difference between cortical and cancellous bone, which results in the screw perforating the pedicle rather than running along the cortex of the pedicle, which also goes unnoticed due to the lack of haptic feedback^36,43^. On the other hand, screw placement is simplified by the absence of soft tissue and bleeding, which can hamper the surgeon even in open procedures^20,44^. While the intra-rater reliability for the exact grading of the screws according to the Neo-classification was only fair for both imaging modalities, the differentiation between acceptable and unacceptable screws seems to be possible with both iCT and CBCT. Possible reasons for the low level of intra-rater agreement include the low contrast of the spine models used in 3D imaging compared to human bone, as well as the screw-to-pedicle diameter ratio of approximately 1 for all levels except C7, which makes it difficult to assess screw perforations with millimeter accuracy.

## Conclusions

Under experimental conditions, high accuracy rates of over > 98% were achieved for navigated cervical pedicle screw placement regardless of the imaging modality or navigation system used despite the high screw-to-pedicle diameter ratio. Radiation doses were significantly lower for CBCT-guided navigation compared to iCT-guided navigation. Further studies on human specimen should be performed under comparable conditions to confirm the results presented.

## Data Availability

All data and statistics are available on request from the corresponding author.

## References

[1] Abumi, K., Itoh, H., Taneichi, H. & Kaneda, K. Transpedicular screw fixation for traumatic lesions of the middle and lower cervical spine: description of the techniques and preliminary report. *J. Spinal Disord*. **7**, 19–28. 10.1097/00002517-199407010-00003 (1994). 8186585 10.1097/00002517-199407010-00003

[2] Jung, Y. G., Lee, S., Jeong, S. K., Kim, M. & Park, J. H. Subaxial cervical pedicle screw in traumatic spinal surgery. *Korean J. Neurotrauma*. **16**, 18–27. 10.13004/kjnt.2020.16.e13 (2020). 32395448 10.13004/kjnt.2020.16.e13 PMC7192805

[3] Tukkapuram, V. R., Kuniyoshi, A. & Ito, M. A. Review of the historical evolution, biomechanical advantage, clinical applications, and safe insertion techniques of cervical pedicle screw fixation. *Spine Surg. Relat. Res. ***3**, 126–135. 10.22603/ssrr.2018-0055 (2019). 31435564 10.22603/ssrr.2018-0055 PMC6690082

[4] Ito, Z. et al. Pedicle screws can be 4 times stronger than lateral mass screws for insertion in the midcervical spine: a biomechanical study on strength of fixation. *J. Spinal Disord Tech. ***27**, 80–85. 10.1097/BSD.0b013e31824e65f4 (2014). 22373932 10.1097/BSD.0b013e31824e65f4

[5] Jones, E. L., Heller, J. G., Silcox, D. H. & Hutton, W. C. Cervical pedicle screws versus lateral mass screws. Anatomic feasibility and biomechanical comparison. *Spine (Phila Pa. 1976) ***22**, 977–982. 10.1097/00007632-199705010-00009 (1997). 9152447 10.1097/00007632-199705010-00009

[6] Coric, D. et al. Navigated minimally invasive posterior cervical pedicle screw fixation. *Int. J. Spine Surg. ***14**, S14–s21. 10.14444/7122 (2020). 33122188 10.14444/7122 PMC7735464

[7] Karaikovic, E. E., Daubs, M. D., Madsen, R. W. & Gaines, R. W. Jr. Morphologic characteristics of human cervical pedicles. *Spine (Phila Pa. 1976) ***22**, 493–500. 10.1097/00007632-199703010-00005 (1997). 9076880 10.1097/00007632-199703010-00005

[8] Tomasino, A. et al. The vertebral artery and the cervical pedicle: morphometric analysis of a critical neighborhood. *J. Neurosurg. Spine ***13**, 52–60. 10.3171/2010.3.Spine09231 (2010). 20594018 10.3171/2010.3.SPINE09231

[9] Amhaz-Escanlar, S., Jorge-Mora, A., Jorge-Mora, T. & Febrero-Bande, M. Diez-Ulloa, M. A. Proposal for a new trajectory for subaxial cervical lateral mass screws. *Eur. Spine J. ***27**, 2738–2744. 10.1007/s00586-018-5670-5 (2018). 29926212 10.1007/s00586-018-5670-5

[10] Hey, H. W. D., Zhuo, W. H., Tan, Y. H. J. & Tan, J. H. Accuracy of freehand pedicle screws versus lateral mass screws in the subaxial cervical spine. *Spine Deform*. **8**, 1049–1058. 10.1007/s43390-020-00119-z (2020). 32314180 10.1007/s43390-020-00119-z

[11] Soliman, M. A. R. et al. Complications associated with subaxial placement of pedicle screws versus lateral mass screws in the cervical spine: systematic review and meta-analysis comprising 1768 patients and 8636 screws. *Neurosurg. Rev. ***45**, 1941–1950. 10.1007/s10143-022-01750-2 (2022). 35138485 10.1007/s10143-022-01750-2

[12] Munusamy, T., Thien, A., Anthony, M. G., Bakthavachalam, R. & Dinesh, S. K. Computed tomographic morphometric analysis of cervical pedicles in a multi-ethnic Asian population and relevance to subaxial cervical pedicle screw fixation. *Eur. Spine J. ***24**, 120–126. 10.1007/s00586-014-3526-1 (2015). 25155836 10.1007/s00586-014-3526-1

[13] Tan, S. H., Teo, E. C. & Chua, H. C. Quantitative three-dimensional anatomy of cervical, thoracic and lumbar vertebrae of Chinese singaporeans. *Eur. Spine J. ***13**, 137–146. 10.1007/s00586-003-0586-z (2004). 14673715 10.1007/s00586-003-0586-z PMC3476578

[14] Shin, E. K., Panjabi, M. M., Chen, N. C. & Wang, J. L. The anatomic variability of human cervical pedicles: considerations for transpedicular screw fixation in the middle and lower cervical spine. *Eur. Spine J. ***9**, 61–66. 10.1007/s005860050011 (2000). 10766079 10.1007/s005860050011 PMC3611356

[15] Farooque, K. et al. Computerized tomography-based morphometric analysis of subaxial cervical spine pedicle in asymptomatic Indian population. *Int. J. Spine Surg. ***12**, 112–120. 10.14444/5017 (2018). 30276069 10.14444/5017 PMC6159543

[16] Lee, D. H. et al. Optimal entry points and trajectories for cervical pedicle screw placement into subaxial cervical vertebrae. *Eur. Spine J. ***20**, 905–911. 10.1007/s00586-010-1655-8 (2011). 21475996 10.1007/s00586-010-1655-8 PMC3099155

[17] Bertram, U. et al. Intraoperative computed tomography-assisted spinal navigation in dorsal cervical instrumentation: a prospective study on accuracy regarding different pathologies and screw types. *World Neurosurg. ***149**, e378–e385. 10.1016/j.wneu.2021.02.014 (2021). 33578024 10.1016/j.wneu.2021.02.014

[18] Mahesh, B., Upendra, B. & Raghavendra, R. Acceptable errors with evaluation of 577 cervical pedicle screw placements. *Eur. Spine J. ***29**, 1043–1051. 10.1007/s00586-020-06359-x (2020). 32152697 10.1007/s00586-020-06359-x

[19] Huang, D. et al. The security analysis of transpedicular screw fixation in the lower cervical spine and a case report. *Spine (Phila Pa)* 36, E1702-1708, (1976). 10.1097/BRS.0b013e31821a5240 (2011). 10.1097/BRS.0b013e31821a5240 22138783

[20] Ishikawa, Y. et al. Intraoperative, full-rotation, three-dimensional image (O-arm)-based navigation system for cervical pedicle screw insertion. *J. Neurosurg. Spine*. **15**, 472–478. 10.3171/2011.6.Spine10809 (2011). 21761967 10.3171/2011.6.SPINE10809

[21] Jung, Y. G. et al. The subaxial cervical pedicle screw for cervical spine diseases: the review of technical developments and complication avoidance. *Neurol. Med. Chir. (Tokyo)*. **60**, 231–243. 10.2176/nmc.ra.2019-0189 (2020). 32295984 10.2176/nmc.ra.2019-0189 PMC7246229

[22] Park, J. H., Jeon, S. R., Roh, S. W., Kim, J. H. & Rhim, S. C. The safety and accuracy of freehand pedicle screw placement in the subaxial cervical spine: a series of 45 consecutive patients. *Spine (Phila Pa)*. 39, 280–285, (1976). 10.1097/brs.0000000000000133 (2014). 10.1097/BRS.0000000000000133 24299725

[23] Mahmoud, A. et al. Cervical spine pedicle screw accuracy in fluoroscopic, navigated and template guided systems-s systematic review. *Tomography ***7**, 614–622. 10.3390/tomography7040052 (2021). 34698301 10.3390/tomography7040052 PMC8544736

[24] Tarawneh, A. M., Haleem, S., D’Aquino, D. & Quraishi, N. The comparative accuracy and safety of fluoroscopic and navigation-based techniques in cervical pedicle screw fixation: systematic review and meta-analysis. *J. Neurosurg. Spine* 1–8. 10.3171/2020.11.Spine201877 (2021). 10.3171/2020.11.SPINE201877 34144517

[25] Shuman, W. H. et al. Intraoperative Navigation in spine surgery: effects on complications and reoperations. *World Neurosurg. ***160**, e404–e411. 10.1016/j.wneu.2022.01.035 (2022). 35033690 10.1016/j.wneu.2022.01.035

[26] Bratschitsch, G. et al. Radiation exposure of patient and operating room personnel by fluoroscopy and navigation during spinal surgery. *Sci. Rep. ***9**, 17652. 10.1038/s41598-019-53472-z (2019). 31776364 10.1038/s41598-019-53472-z PMC6881318

[27] Helm, P. A., Teichman, R., Hartmann, S. L. & Simon, D. Spinal navigation and imaging: history, trends, and future. *IEEE Trans. Med. Imaging ***34**, 1738–1746. 10.1109/tmi.2015.2391200 (2015). 25594965 10.1109/TMI.2015.2391200

[28] Neo, M., Sakamoto, T., Fujibayashi, S. & Nakamura, T. The clinical risk of vertebral artery injury from cervical pedicle screws inserted in degenerative vertebrae. *Spine (Phila Pa. 1976) ***30**, 2800–2805. 10.1097/01.brs.0000192297.07709.5d (2005). 16371908 10.1097/01.brs.0000192297.07709.5d

[29] Hwang, Y. S., Tsai, H. Y., Lin, Y. Y. & Lui, K. W. Investigations of organ and effective doses of abdominal cone-beam computed tomography during transarterial chemoembolization using Monte Carlo simulation. *BMC Med. Imaging ***18**10.1186/s12880-018-0247-7 (2018). 10.1186/s12880-018-0247-7 PMC5800092 29402236

[30] Chachan, S., Abd Razak, B., Loo, H. R. & Allen, W. L. Shree Kumar, D. Cervical pedicle screw instrumentation is more reliable with O-arm-based 3D navigation: analysis of cervical pedicle screw placement accuracy with O-arm-based 3D navigation. *Eur. Spine J. ***27**, 2729–2736. 10.1007/s00586-018-5585-1 (2018). 29651593 10.1007/s00586-018-5585-1

[31] Coric, D. & Rossi, V. Percutaneous posterior cervical pedicle instrumentation (C1 to C7) with navigation guidance: early series of 27 cases. *Global Spine J. ***12**, 27s–33s. 10.1177/21925682211029215 (2022). 35393883 10.1177/21925682211029215 PMC8998482

[32] Farber, S. H. et al. Accuracy of subaxial cervical pedicle screw placement using direct visualization versus computed tomography–based navigation. *Clin. Spine Surg. ***35**, E104–E110. 10.1097/bsd.0000000000001141 (2022). 34379611 10.1097/BSD.0000000000001141

[33] Zhang, H. L., Zhou, D. S. & Jiang, Z. S. Analysis of accuracy of computer-assisted navigation in cervical pedicle screw installation. *Orthop. Surg. ***3**, 52–56. 10.1111/j.1757-7861.2010.00110.x (2011). 22009981 10.1111/j.1757-7861.2010.00110.x PMC6583217

[34] Gertzbein, S. D. & Robbins, S. E. Accuracy of pedicular screw placement in vivo. *Spine ***15**, 11–14 (1990). 2326693 10.1097/00007632-199001000-00004

[35] Heo, Y. et al. The learning curve of subaxial cervical pedicle screw placement: how can we avoid neurovascular complications in the initial period? *Oper. Neurosurg. (Hagerstown) ***17**, 603–607. 10.1093/ons/opz070 (2019). 31173103 10.1093/ons/opz070

[36] Frisk, H. et al. Feasibility and accuracy of thoracolumbar pedicle screw placement using an augmented reality head mounted device. *Sens. (Basel) ***22**10.3390/s22020522 (2022). 10.3390/s22020522 PMC8779462 35062483

[37] Miller, C. A., Ledonio, C. G., Hunt, M. A., Siddiq, F. & Polly, D. W. Jr. Reliability of the planned pedicle screw trajectory versus the actual pedicle screw trajectory using intra-operative 3D CT and image guidance. *Int. J. Spine Surg. ***10**10.14444/3038 (2016). 10.14444/3038 PMC5130325 27909659

[38] Beisemann, N. et al. Radiation exposure for pedicle screw placement with three different navigation system and imaging combinations in a sawbone model. *BMC Musculoskelet. Disord*. **24**, 752. 10.1186/s12891-023-06880-2 (2023). 37742007 10.1186/s12891-023-06880-2 PMC10517448

[39] Baumgart, L., Ille, S., Kirschke, J. S., Meyer, B. & Krieg, S. M. Radiation doses and accuracy of navigated pedicle screw placement in cervical and thoracic spine surgery: a comparison of sliding gantry CT and mobile cone-beam CT in a homogeneous cohort. *J. Neurosurg. Spine ***39**, 363–369. 10.3171/2023.4.Spine23174 (2023). 37310023 10.3171/2023.4.SPINE23174

[40] Klingler, J. H. et al. Patient radiation exposure from intraoperative computed tomography in spinal surgery. *Spine J. ***22**, 1576–1578. 10.1016/j.spinee.2022.03.008 (2022). 35351665 10.1016/j.spinee.2022.03.008

[41] Tkatschenko, D. et al. Navigated percutaneous versus open pedicle screw implantation using intraoperative CT and robotic cone-beam CT imaging. *Eur. Spine J. ***29**, 803–812. 10.1007/s00586-019-06242-4 (2020). 31820094 10.1007/s00586-019-06242-4

[42] Foster, N. et al. Image quality and dose comparison of 3 mobile intraoperative three-dimensional imaging systems in spine surgery. *World Neurosurg.* (2022). 10.1016/j.wneu.2021.12.103 10.1016/j.wneu.2021.12.103 PMC9006663 34979287

[43] Beisemann, N. et al. Comparison of three imaging and navigation systems regarding accuracy of pedicle screw placement in a sawbone model. *Sci. Rep. ***12**, 12344. 10.1038/s41598-022-16709-y (2022). 35853991 10.1038/s41598-022-16709-y PMC9296669

[44] Uehara, M. et al. Screw perforation features in 129 consecutive patients performed computer-guided cervical pedicle screw insertion. *Eur. Spine J. ***23**, 2189–2195. 10.1007/s00586-014-3502-9 (2014). 25095759 10.1007/s00586-014-3502-9

